# Comparative Efficacies of Antimicrobial Catheter Lock Solutions for Fungal Biofilm Eradication in an in Vitro Model of Catheter-Related Fungemia

**DOI:** 10.3390/jof3010007

**Published:** 2017-02-10

**Authors:** Joel Rosenblatt, Ruth A. Reitzel, Nylev Vargas-Cruz, Anne-Marie Chaftari, Ray Hachem, Issam I. Raad

**Affiliations:** Department of Infectious Diseases, Infection Control & Employee Health, University of Texas MD Anderson Cancer Center, Houston, TX 77030, USA; RReitzel@mdanderson.org (R.A.R.); NSVargas@mdanderson.org (N.V.-C.); AChaftar@mdanderson.org (A.-M.C.); RHachem@mdanderson.org (R.H.); IRaad@mdanderson.org (I.I.R.)

**Keywords:** catheter bloodstream infection, *Candida*, antimicrobial, catheter lock solution

## Abstract

Fungal catheter-related bloodstream infections (CRBSIs)—primarily due to *Candida* species—account for over 12% of all CRBSIs, and have been progressively increasing in prevalence. They present significant health and economic burdens, and high mortality rates. Antimicrobial catheter lock solutions are an important prophylactic option for preventing fungal CRBSIs. In this study, we compared the effectiveness of two FDA-approved catheter lock solutions (heparin and saline) and three experimental antimicrobial catheter lock solutions—30% citrate, taurolidine-citrate-heparin (TCH), and nitroglycerin-citrate-ethanol (NiCE)—in an in vitro model of catheters colonized by fungi. The fungi tested were five different strains of *Candida* clinical isolates from cancer patients who contracted CRBSIs. Time-to-biofilm-eradication was assessed in the model with 15, 30, and 60 min exposures to the lock solutions. Only the NiCE lock solution was able to fully eradicate all fungal biofilms within 60 min. Neither 30% citrate nor TCH was able to fully eradicate any of the *Candida* biofilms in this time frame. The NiCE lock solution was significantly superior to TCH in eradicating biofilms of five different *Candida* species (*p* = 0.002 for all).

## 1. Introduction

Central line associated bloodstream infections (CLABSIs) are a significant public health problem in the US reportedly affecting 250,000 patients annually, causing approximately 30,000 mortalities, and having an associated economic burden estimated to be $45,000 per patient [1,2]. The epidemiology and source of CLABSIs in the US has been continuously evolving over the last two decades. The adoption of precautionary procedural bundles during insertion and infusion procedures has decreased external surface-sourced CLABSIs [3,4]. Precautionary procedural bundles include rigorous hand hygiene, the use of full-length sterile drapes, insertion-site skin antisepsis with chlorhexidine, and regular assessment of whether catheter removal is warranted. Despite this progress in reducing CLABSIs, those that do occur are increasingly luminally-sourced, and the catheter lumen is now implicated as the source of CLABSI in as much as 90% [5,6] of all CLABSIs. In parallel, there has been a shift from a predominance of Gram-positive CLABSIs to increasing proportions of Gram-negative and fungal CLABSIs. In the 1990s, the NNIS (National Nosocomial Infection Surveillance System) reported that 8% of CLABSIs in the US were of fungal derivation (primarily *Candida albicans*, which caused 62.5% of candidemias) [7]. In 2006, the NHSN (National Healthcare Safety Network) reported that 11.8% of CLABSIs in the US were candidemias (50% due to *Candida albicans*) [8], and in 2010, the prevalence of candidemia CLABSIs increased to 14.6% (44% due to *Candida albicans*) [9]. This increase in prevalence was accompanied by changes in the variety of *Candida* species causing these bloodstream infections. The proportion of catheter-related candidemias caused by *Candida albicans* has declined, while there has been a concomitant increase in the prevalence of candidemias caused by *Candida parapsilosis*, *Candida kruseii*, *Candida tropicalis*, and most notably *Candida glabarata* and azole-resistant *Candida glabarata* [10]. Possible reasons for the epidemiological shift in causative *Candida* species are improvements in diagnostic methodologies for the detection of *non-albicans Candida* species, as well as the emergence of resistant *non-albicans Candidas* [11]. CLABSI caused by *Candida* spp. have high mortality rates of >40% [12,13]; hence, there is a strong need for improved measures to prevent these infections. 

In response to the increased prevalence of luminally-sourced CLABSIs, new technologies have also been introduced to protect luminal surfaces from microbial colonizations that ultimately cause bloodstream infections. Antimicrobial swabs and caps have been introduced that disinfect the luer connectors of catheters [14]. Nevertheless, most of the luminal surfaces remain unprotected. Antimicrobial catheter coatings have also been introduced, but antibiotic coatings which have been notably successful in reducing the incidence of CLABSIs have had limited effectiveness against *Candida* species [15]. Antiseptic coatings based on silver and chlorhexidine have very limited antimicrobial durability, and therefore have largely failed to significantly reduce the incidence of CLABSIs in well-controlled clinical trials [15]. Hence, as uses and indwell durations of central lines continue to increase, there remains a significant unmet need for more potent and more durable protection of catheter lumens against microbial colonization, including *Candida* species colonization that is a prelude to central line-associated candidemia.

Antimicrobial catheter lock solutions are one promising emerging prophylactic technology [16] with the potential to reduce and prevent *Candida* colonization of catheter lumens. Catheter lumens need to be hydraulically locked between uses to prevent the introduction of air emboli into the bloodstream. Therefore, antimicrobial catheter lock solutions provide an appealing approach to prevent luminally-sourced CLABSIs, since they are repetitively instilled and fill catheter lumens on a frequent basis. The most widely used catheter lock solutions are saline and heparin, which maintain hydraulic patency of catheters, and—in the case of heparin—potentially add protection against thrombotic occlusion of catheter lumens [17]. The lock solutions, however, provide little (if any) protection against microbial colonization, and there is evidence that heparin can actually promote microbial attachment to catheter surfaces [18]. In many cases, a few colonies of microbes can be introduced to catheter lumens during handling and infusions, which then begin to attach, grow, colonize, and form biofilms. Therefore, to be truly effective, a prophylactic antimicrobial catheter lock solution needs to be capable of eradicating biofilms that might have formed on catheter surfaces during intermittent uses [19,20]. Like antibiotic lock solutions, antifungal lock solutions [21] have been used; however, the risk of developing drug-resistant fungal pathogens discourages their prophylactic use. Hence there is a need for non-antibiotic and non-antifungal antimicrobial lock solutions that can be used preventatively without encouraging the development of resistant pathogens. This need is particularly urgent in the US, where no non-antibiotic and non-antifungal antimicrobial catheter lock solutions are currently approved. Two types of non-antibiotic and non-antifungal catheter lock solutions—concentrated citrate and taurolidine-based lock solutions—have been approved in the European Union (EU). Additionally, the development and clinical assessment of an additional type of non-antibiotic and non-antifungal antimicrobial catheter lock solution has been recently reported in the US (nitroglycerin-citrate-ethanol catheter lock solution) [22,23,24]. Here we report results from an in vitro study in a well-established biofilm eradication model [25] for microbial colonization of catheter surfaces that comparatively assessed the potency of these three non-antifungal antimicrobial lock solutions (currently either approved in the EU or undergoing active clinical development in the US) for eradicating biofilms formed on catheter surfaces by *Candida* species.

## 2. Materials and Methods

### 2.1. Lock Solutions

Lock solutions prepared for testing were 1.35% taurolidine + 3.5% citrate + 1000 U/mL heparin (TCH), 30% citrate, 0.9% saline (saline), 200 Units/mL heparin (heparin), and 0.003% nitroglycerin + 4% citrate + 22% ethanol (NiCE). The 0.003% NiCE was recently assessed in a human clinical trial [26]. All lock solutions except saline were prepared from their individual components, nitroglycerin (Baxter Healthcare Corporation, Deerfield, IL, USA), disodium citrate dehydrate (Sigma-Aldrich, St. Louis, MO, USA), ethanol (Sigma-Aldrich), taurolidine (Toronto Research Chemicals, North York, ON, USA), and heparin (Sagent Pharmaceuticals, Schaumberg, IL, USA). Prefilled syringes of saline lock solution were purchased (Becton, Dickinson and Company, Franklin Lakes, NJ, USA). The 200 U/mL heparin concentration was selected for the control heparin lock solution because that concentration is typically used in our hospital for heparin locking. The final concentrations of the lock solutions used in antimicrobial testing were 0.003% nitroglycerin + 4% citrate + 22% ethanol (NiCE), 1.35% taurolidine + 3.5% citrate + 1000 U/mL heparin (TCH), 30% citrate, 0.9% saline, and 200 U/mL heparin. 

### 2.2. Fungal Strains

A broad spectrum of highly virulent, biofilm-forming, fungal (yeast) pathogens from our hospital were used for testing. These strains included *Candida albicans* (CA, MDA 117), *Candida glabrata* (CG, MDA 115), *Candida tropicalis* (CT, MDA 112), *Candida kruseii* (CK, MDA116), and *Candida parapsilosis* (CP, MDA 113). These pathogens were clinical isolates selected from the MD Anderson Infectious Disease lab stored bank of organisms from cancer patient cultures that are routinely used for biofilm colonization/eradication model testing. Fresh organisms were grown on Sabrououd dextrose agar from glycerol stock overnight at 37 °C. For testing, pure culture was inoculated into Muller Hinton Broth (MHB) and diluted to 0.5 McFarland. Additional dilutions were made as necessary for testing. All culture reagents were purchased from Fisher Scientific (Waltham, MA, USA).

### 2.3. Assessment of Time to Biofilm Eradication

In vitro antimicrobial assessments of NiCE lock compared to commercially-available chelator and standard of care lock solutions were conducted using an established time-to-kill biofilm eradication model—the modified Kuhn’s model [25]. Briefly, 1 cm diameter silicone discs were placed into a 24-well tissue culture plate and incubated with donor human plasma at 37 °C for 24 h. The plasma was then removed, replaced with 1 mL of 5.5 × 10^5^ CFU of yeast inocula, and incubated at 37 °C for an additional 24 h. Inoculum was removed, and the discs were washed shaking at 100 rpm for 30 min in 0.9% sterile saline in order to remove any non-adherent organisms. After washing, discs were exposed to 1 mL various lock solutions and incubated at 37 °C for 15, 30, or 60 min. Saline flush solution was used a negative control. Subsequently, discs were removed and placed in 5 mL of 0.9% sterile saline and sonicated (60 Hz and 150 W) for 15 min to disrupt any remaining biofilm. Resulting solutions were quantitatively cultured by serial dilution in 0.9% sterile saline and plating 100 µL onto Sabouraud dextrose agar. All biofilm eradication experiments were performed with six replicates.

### 2.4. Statistical Analyses

The Kruskal–Wallis test was used to determine whether there was a significant difference in the medians in any of the lock solutions tested. Pairwise comparisons were assessed using the Mann–Whitney *U* test to compare performance of NiCE, TCH, and control saline lock solutions at the 60 min time point. All tests were two-sided with an alpha level of 0.05. A *p-*value less than 0.05 (*p* < 0.05) was utilized to determine statistical significance. 

## 3. Results

### 3.1. Time to Biofilm Eradication

**Figure 1:**
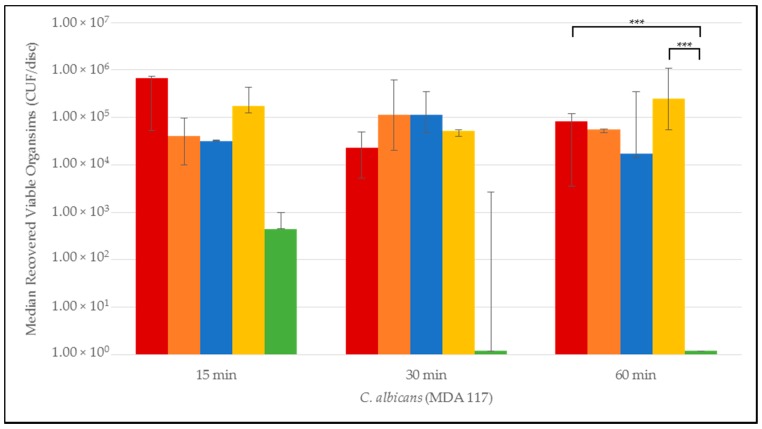
Time-to-kill eradication of *Candida albicans* (CA) biofilm—NiCE (0.003% nitroglycerin + 4% citrate + 22% ethanol) lock solution eradicated CA biofilm within 30 min to 60 min. None of the other lock solutions tested fully eradicated biofilm by the 60 min timepoint. A significant difference (*p* = 0.002) is denoted with *** between 0.003% NiCE when compared to both TCH (1.35% taurolidine + 3.5% citrate + 1000 U/mL heparin) and 0.9% saline control. Graph Key: • 0.9% saline; • 200 U heparin; • 30% citrate; • 1.35% taurolidine + 3.5% citrate + 1000 U heparin; • 0.003% nitroglycerin + 4% citrate + 22% ethanol.

**Figure 2:**
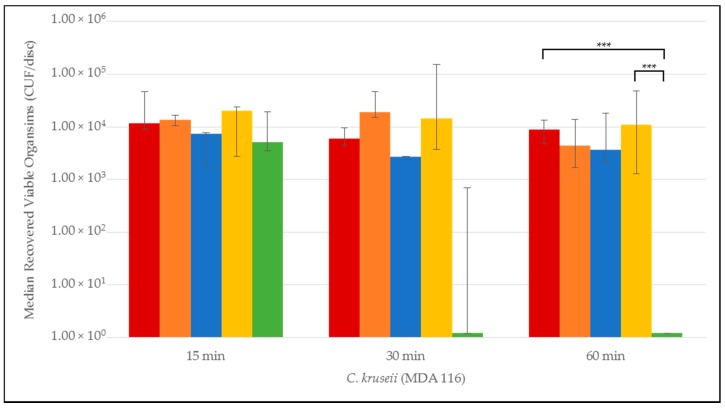
Time-to-kill eradication of *Candida kruseii* (CK) biofilm—NiCE lock solution eradicated CK biofilm within 30 min. None of the other lock solutions tested fully eradicated biofilm by the 60 min timepoint. A significant difference (*p* = 0.002) is denoted with *** between 0.003% NiCE when compared to both TCH and 0.9% saline control. Graph Key: • 0.9% saline; • 200 U heparin; • 30% citrate; • 1.35% taurolidine + 3.5% citrate + 1000 U heparin; • 0.003% nitroglycerin + 4% citrate + 22% ethanol.

**Figure 3:**
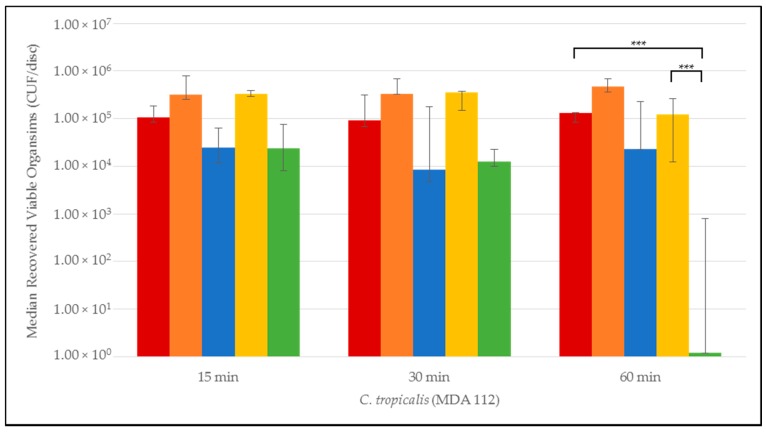
Time-to-kill eradication of *Candida tropicalis* (CT) biofilm—NiCE lock solution eradicated CT biofilm within 1 h. None of the other lock solutions tested fully eradicated biofilm by the 60 min timepoint. A significant difference (*p* = 0.002) is denoted with *** between 0.003% NiCE when compared to both TCH and 0.9% saline control. Graph Key: • 0.9% saline; • 200 U heparin; • 30% citrate; • 1.35% taurolidine + 3.5% citrate + 1000 U heparin; • 0.003% nitroglycerin + 4% citrate + 22% ethanol.

**Figure 4:**
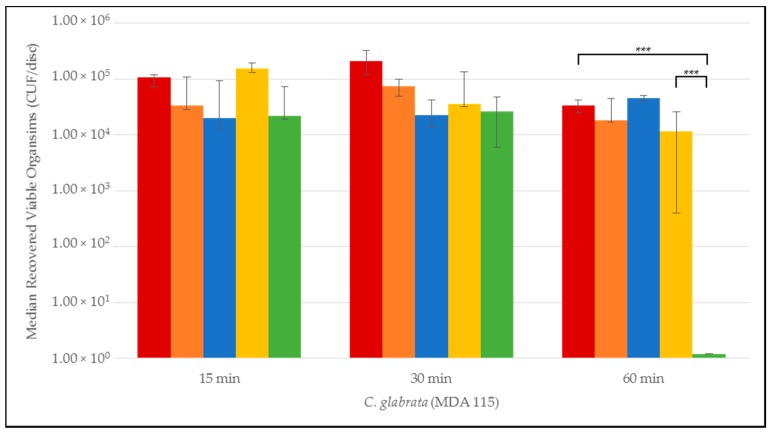
Time-to-kill eradication of *Candida glabrata* (CG) biofilm—NiCE lock solution eradicated CG biofilm within 1 h. None of the other lock solutions tested fully eradicated biofilm by the 60 min timepoint. A significant difference (*p* = 0.002) is denoted with *** between 0.003% NiCE when compared to both TCH and 0.9% saline control. Graph Key: • 0.9% saline; • 200 U heparin; • 30% citrate; • 1.35% taurolidine + 3.5% citrate + 1000 U heparin; • 0.003% nitroglycerin + 4% citrate + 22% ethanol.

**Figure 5:**
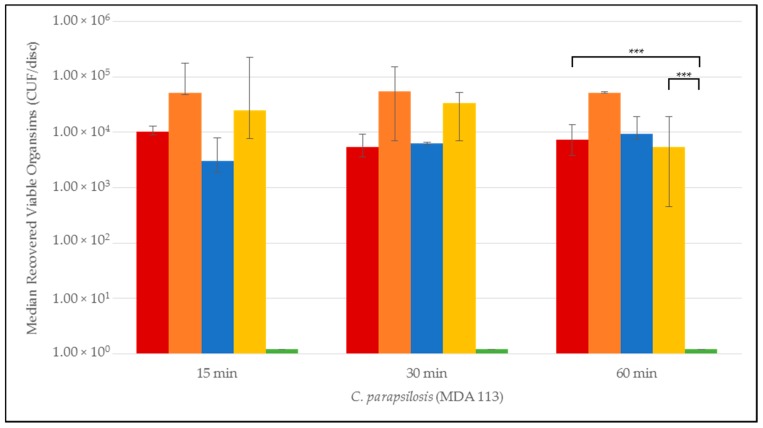
Time-to-kill eradication of *Candida parapsilosis* (CP) biofilm—NiCE lock solution eradicated CP biofilm within 15 min to 30 min. None of the other lock solutions tested fully eradicated biofilm by the 60 min timepoint. A significant difference (*p* = 0.002) is denoted with *** between 0.003% NiCE when compared to both TCH and 0.9% saline control. Graph Key: • 0.9% saline; • 200 U heparin; • 30% citrate; • 1.35% taurolidine + 3.5% citrate + 1000 U heparin; • 0.003% nitroglycerin + 4% citrate + 22% ethanol.

Quantitative time-to-eradication results for the different *Candida* strains tested are presented in Figure 1, Figure 2, Figure 3, Figure 4 and Figure 5. The median number and range of viable organisms recovered from biofilm of a specific *Candida* species subjected to lock exposures of 15, 30, and 60 min is presented in each figure. For *Candida albicans* biofilms (Figure 1), the NiCE lock solution demonstrated complete eradication of the biofilm within 15–30 min exposure. The other lock solutions demonstrated little to no reduction in viable organism content in *Candida albicans* biofilms over the time frames of exposure. The NiCE lock solution was able to fully eradicate *Candida kruseii* biofilms within 15–30 min exposure (Figure 2). The other lock solutions tested demonstrated little-to-no reduction in viable organism content of *Candida kruseii* biofilms following 60 min exposure. Against *Candida tropicalis* (Figure 3) and *Candida glabarata* biofilms (Figure 4), NiCE lock solution fully eradicated viable organisms within 30–60 min of exposure. The other lock solutions tested demonstrated little to no reduction in viable organisms embedded in biofilms for these strains following 60 min exposure. Against *Candida parapsilosis* biofilms (Figure 5), NiCE lock solution fully eradicated viable organisms within 15 min of exposure. The other lock solutions tested demonstrated little to no reduction in the viable organism content of *Candida parapsilosis* biofilms following up to 60 min of exposure. In some cases, heparin-containing lock solutions demonstrated a slight tendency towards increasing median viable organism concentrations in biofilms with the brief exposures tested here, but these increases were not statistically significant. 

### 3.2. Statistical Comparison of Antimicrobial Activity of NiCE and Taurolidine + Citrate + Heparin Lock Solutions

NiCE solutions fully eradicated biofilms of all strains of *Candida* tested within 60 min. Among all lock solutions tested, there was a significant difference between NiCE and any of the groups at 60 min (*p* < 0.001) for all strains. Further *ad hoc* pairwise comparisons of NiCE or TCH compared to 0.9% saline negative controls showed that NiCE was statistically significant compared to saline for all organisms tested (*p* = 0.002). Compared to saline control, TCH was only statistically significant for *Candida glabrata* (*p* = 0.004); all other strains were non-significant. The relative reductions of NiCE versus TCH lock solution were significant (*p* = 0.002) for all strains of *Candida* tested.

## 4. Discussion

In this study, we evaluated catheter lock solutions that were either available for clinical use or in active clinical development (therefore with the near-term potential to impact clinical practice) for potency in eradicating *Candida* biofilms. Several lock solutions tested contained citrate in concentrations ranging from 3.5% to 30% (30% citrate, TCH and NiCE). For the brief exposures tested here, the 30% citrate lock solution demonstrated little to no reduction in fungal biofilm viability. Thus, it appears that citrate by itself is unable to rapidly eradicate fungal biofilms. It is possible that citrate alone would exert more significant effects on fungal biofilm viability with much longer exposures; however, fungal pathogens appear able to survive metal ion deprivation by chelation for brief durations. Chelators such as citrate in combination with other antimicrobial agents have been reported to synergistically contribute anti-biofilm effects, and may play contributory antimicrobial roles in TCH and NiCE lock solutions [27]. 

Two lock solutions tested contained heparin in concentrations ranging from 200–1000 units/mL (heparin and TCH). Neither lock solution demonstrated significant reduction in fungal organism concentrations in biofilms following brief exposures. There was a weak but not significant trend to increased viable organism concentrations in some of the heparin-containing lock solutions. The ability of heparin to stimulate biofilm formation for fungal and bacterial organisms has been previously demonstrated with longer exposures [28]; however, it is not possible to make conclusions about the stimulatory effect of brief exposures to heparin for fungal organisms in this study. Brief exposures of fungal biofilms to heparin in this study demonstrated little-to-no potential for reducing viable fungal organism concentrations. 

Taurolidine-containing lock solutions have been assessed in vitro as well as in several clinical studies. Taurolidine has multiple mechanisms of biological activity; its antimicrobial activity is reportedly due to the reaction of methylol and methylene iminium metabolites with cell wall and cytoplasmic constituents [29,30]. Antifungal activity against planktonic *Candida albicans* was reported in vitro at 1.35% concentration, but not at 0.675% concentration, and only after 24 h exposure [31]. Following 72 h exposure of *Candida albicans* biofilms, 1.35% taurolidine lock solution was not able to demonstrate a significant reduction in viable fungal organism concentration versus heparin lock solution [31]. The extracellular matrix of *Candida* biofilms is rich in polysaccharides [32], and taurolidine is reported to bind to polysaccharides [33,34]. This may in part cause some attenuation of its antifungal activity against biofilm. Clinical testing of vascular catheters with indwells ranging from 51–590 days demonstrated that about 88% of the catheters locked with taurolidine lock solution had intraluminal colonization with a median coverage of 80% of the luminal surfaces [35]. This finding is consistent with the in vitro results of this study in that once biofilm forms, taurolidine appears to be incapable of rapidly eradicating it, although it might still be able to moderate pathogenicity [35]. 

The NiCE lock solution was the only ethanol and nitroglycerin-containing lock solution tested. Previous studies have shown this combination to possess synergistic antimicrobial activity [36]. The synergy was hypothesized to derive from the antimicrobial contributions of nitric oxide (metabolic derivative of nitroglycerin), the denaturing effect of ethanol, and the chelating activity of citrate. Both nitroglycerin [37] and ethanol [38] have previously demonstrated antifungal activity. The relative contribution of nitroglycerin in the NiCE combination was demonstrated by conducting experiments with half the nitroglycerin (0.0015%) that was present in the NiCE lock solution that was clinically assessed [26]. At this reduced nitroglycerin concentration, eradication of the *Candida albicans* biofilm required greater than 30 min (was completed within 60 min versus within 30 min for the 0.003% concentration), and eradication of the *Candida parapsilosis* biofilm required greater than 15 min (was completed within 30 min versus within 15 min for the 0.003% concentration). Both nitroglycerin and ethanol are small uncharged molecules with some lipophilicity, which are properties that likely aid in rapid and invasive biofilm penetration. From a safety standpoint, the 22% ethanol concentration is below the 28% threshold required for precipitation of proteins in human blood [39]. This concentration is also well below ethanol concentrations (greater than 40%) in lock solutions that potentially induced catheter mechanical function impairment or transient patient inebriation effects in previous studies [40]. A recent clinical trial for NiCE lock solution [26] containing 22% ethanol in 60 patients demonstrated no impairment of catheter hydraulic function or significant patient adverse reactions following repeated locking and flushing cycles. The same study also showed no clinically significant hypotensive effects of patient exposure to the doses of nitroglycerin in NiCE (which causes vasodilation at higher doses) [41]. Results here demonstrated that in combination at the concentrations of the NiCE lock solution, the combination was able to eradicate biofilms for all fungal organisms tested. 

Biofilm colonization of vascular catheters is a prelude to catheter-related bloodstream infections [42]. The prevalence of catheter-related candidemias has increased, as have the diversity of *Candida* strains involved in catheter-related candidemias. Additionally, catheter-related candidemia is associated with high morbidity and mortality, and therefore improved prevention measures remain a significant medical need. Antimicrobial lock solutions are an attractive approach to intraluminal colonization of vascular catheters by fungal biofilms. Since lumens of indwelling vascular catheters are used for many purposes—and some with long infusion durations—they therefore present a multitude of opportunity for microbes to attach and begin colonizing luminal surfaces. Hence, optimal prevention of catheter-related fungal candidemia requires not only inhibition of colonization, but also the ability to rapidly eradicate biofilm that may have formed during intervals that catheter lumens were not filled with an antimicrobial lock solution. Taurolidine-citrate-heparin and 30% citrate antimicrobial lock solutions did not demonstrate rapid (within 60 min) eradication of *C. albicans*, *C. tropicalis*, *C. kruseii*, *C. glabrata*, or *C. parapsilosis* biofilms. Nitroglycerin-citrate-ethanol was the only antimicrobial lock solution tested in this study that could rapidly eradicate all of these fungal biofilms. There were some differences in how quickly different strains of *Candida* biofilms were eradicated. This may be a result of differences in extracellular and cellular matrix composition [11,43] and the presence of different phenotypes (for example hyphal or pseudohyphal) in the different biofilms. The greater anti-fungal potency of nitroglycerin-citrate-ethanol versus taurolidine-citrate-heparin antimicrobial lock solutions was significant. Since neither lock solution contains therapeutic antibiotic or antifungal agents, the potential for clinically-significant development of antifungal resistance is expected to be minimal. Further clinical testing is needed to demonstrate that these results translate to a reduction in catheter-related fungal bloodstream infections.

## 5. Conclusions

Antimicrobial catheter lock solutions not containing antibiotic or antifungal agents that are effective against *Candida* biofilms remain an urgent unmet medical need. Three such lock solutions with near term potential to impact clinical practice were evaluated in this in vitro study of *Candida* biofilm eradication. The NiCE lock solution was able to eradicate biofilms of all five *Candida* strains within 60 min exposures. 30% Citrate and TCH lock solutions were not able to eradicate any of the *Candida* biofilms within 60 min exposures. The antifungal efficacy of NiCE was significantly superior to TCH for all *Candida* strains tested.
